# Sharing of clinical data in a maternity setting: How do paper hand-held records and electronic health records compare for completeness?

**DOI:** 10.1186/s12913-014-0650-x

**Published:** 2014-12-21

**Authors:** Glenda Hawley, Claire Jackson, Julie Hepworth, Shelley A Wilkinson

**Affiliations:** APHCRI Centre of Research Excellence in Primary Health Care Microsystems, School of Medicine, Discipline of General Practice, University of Queensland, Level 8 Health Sciences Building, Building 16/910, Herston, Brisbane, QLD 4029 Australia; Mater Research, Mothers & Babies Theme, Mater Health Services, South Brisbane, 4101 Australia; School of Public Health & Social Work, Queensland University of Technology, Victoria Park Road, Kelvin Grove, QLD 4059 Australia; Department of Nutrition and Dietetics, Mater Health Services, South Brisbane, 4101 Australia

**Keywords:** Maternity, Shared-care, General practitioner (GP), Paper hand-held record (PHR), Electronic health record (EHR), Best Practice Variable

## Abstract

**Background:**

Historically, the paper hand-held record (PHR) has been used for sharing information between hospital clinicians, general practitioners and pregnant women in a maternity shared-care environment. Recently in alignment with a National e-health agenda, an electronic health record (EHR) was introduced at an Australian tertiary maternity service to replace the PHR for collection and transfer of data. The aim of this study was to examine and compare the completeness of clinical data collected in a PHR and an EHR.

**Methods:**

We undertook a comparative cohort design study to determine differences in completeness between data collected from maternity records in two phases. Phase 1 data were collected from the PHR and Phase 2 data from the EHR. Records were compared for completeness of best practice variables collected The primary outcome was the presence of best practice variables and the secondary outcomes were the differences in individual variables between the records.

**Results:**

Ninety-four percent of paper medical charts were available in Phase 1 and 100% of records from an obstetric database in Phase 2. No PHR or EHR had a complete dataset of best practice variables. The variables with significant improvement in completeness of data documented in the EHR, compared with the PHR, were urine culture, glucose tolerance test, nuchal screening, morphology scans, folic acid advice, tobacco smoking, illicit drug assessment and domestic violence assessment *(p = 0.001)*. Additionally the documentation of immunisations (pertussis, hepatitis B, varicella, fluvax) were markedly improved in the EHR *(p = 0.001)*. The variables of blood pressure, proteinuria, blood group, antibody, rubella and syphilis status, showed no significant differences in completeness of recording.

**Conclusion:**

This is the first paper to report on the comparison of clinical data collected on a PHR and EHR in a maternity shared-care setting. The use of an EHR demonstrated significant improvements to the collection of best practice variables. Additionally, the data in an EHR were more available to relevant clinical staff with the appropriate log-in and more easily retrieved than from the PHR. This study contributes to an under-researched area of determining data quality collected in patient records.

**Electronic supplementary material:**

The online version of this article (doi:10.1186/s12913-014-0650-x) contains supplementary material, which is available to authorized users.

## Background

The paper hand-held record (PHR) has been a successful and integral tool used in maternity shared-care for almost sixty years. Hamilton introduced the ‘Co-op (co-operation) card' in 1956 in the United Kingdom (UK) and since this time women and clinicians have used some version of the PHR to record maternity care [1]. The woman carries the PHR and care given is documented at each visit to either the community clinician or the hospital. The benefits of the PHR have been demonstrated in previous, mainly descriptive papers but little formal evaluation has been done on the data collected in the PHR [2].

Increasingly, the use of a patient electronic health record (EHR) has emerged together with evaluations of its implementation in a variety of health settings. Implementation issues of standardising processes, safety and security, promoting evidence based practice, ease of use, easing workload and using less paper charts have all been cited [3]. The EHR is designed to use information in a digital format that can be used by both patients and health care providers from anywhere, at any time [3]. Digital records are accessed using a variety of devices and media, including USB (portable memory) stick and web-enabled interfaces of personal computers, smart phones or tablets.

Access to best practice maternity care is a major priority on the Australian national health agenda and to address the fragmentation of data currently available, a maternity EHR has been developed and is trialled in many sites, including a general practice (GP) shared-care setting [4,5]. Shared-care is seen as a service provided between the primary and secondary care sectors, with GPs as the fundamental central component [6]. The EHR in a maternity shared-care setting aims to improve the integration of clinical care between GPs, midwives, allied health professionals, and women.

A major component of integrating clinical care between these sectors is having significant clinical data available as needed. Having access to valid, reliable and complete information is fundamental to improving patient and health care communication and patient safety. The PHR is still the main source of information in a maternity shared-care environment but as this information is written in a free text form, it may not be retrieved easily if the record is missing or is not accessible for multiple health providers simultaneously. It is not known if the introduction of an EHR will improve access to and completeness and reliability of data or information collected in a maternity record. With an increased emphasis on utilising electronic data and communication systems, the need to know to what extent an EHR will improve the quality of available maternity data is essential. The aim of this study was to describe and compare the completeness of recorded best practice variables in a maternity PHR and the EHR.

## Methods

### Design

The study used a comparative cohort design to determine differences between sets of clinical data collected in two phases. To avoid the possibility of data being collected for the 2 Phases at the same time, Phase 1 data collection was completed before the introduction of the EHR. In consultation with the MH statistician, Phase 2 data were collected 6 months after the introduction of the EHR in 2012.

### Study setting, participants and data sources

The study was completed in a South East Queensland (Australia) tertiary maternity hospital (MH) with an established shared-care arrangement with General Practitioners (GPs). GPs who share maternity care with the MH are ‘aligned’ following an education program coordinated with obstetric advisory consultation. In a GP shared-care arrangement, women visit the MH routinely at ‘booking in’ (~12-16 weeks) and again at the 36–40 week gestation period. The aligned GP manages the care of women at visits between these time periods. During the visits to the antenatal clinic the women are seen by a variety of clinicians, including midwives, obstetricians, and allied health clinicians (e.g. physiotherapists, social workers, dietitians, and psychologists). At the ‘booking in’ visit the woman is seen by a midwife and a hospital based doctor, where physical observations and an antenatal history are taken and documented in an antenatal record. Both the hospital health care providers and the GPs are trained in data entry requirements.

Prior to July 2012, the PHR was the only antenatal record available for use at the MH. After this date, the MH introduced a maternity EHR. Antenatal woman could elect to use a PHR or an EHR to share information between the hospital and her GP. The EHR has a functionality that does not allow progression of data entry if a mandatory field (ie. requires an entry) is left blank.

Eligible data for the study were obtained from the hospital data set pertaining to women who participated in the GP shared-care maternity model of care who were over 18 years of age, able to understand and speak English. The data analysed in Phase 1 of this study were obtained from conducting a chart audit of PHRs used by pregnant women during the period of 01 July 2011 and 31 December 2011. Phase 2 data were extracted from the obstetric database; a repository for antenatal information from the EHR at the MH during the period of 01 January 2013 and 31 June 2013. A comparison of the paper and electronic data systems is seen in Additional file 1. The hospital health care providers and GPs perform the same clinical role in the delivery of care using the PHR and EHR, in this setting. The dataset in both phases were randomly selected, with variables predominantly being collected during the hospital ‘booking in visit’ and additional important variables collected by the GP.

### Outcome variables

Specific evidence-based, best-practice variables (further referred to as ‘best practice variables’) were chosen after examining the National Clinical Practice Guidelines for antenatal care and guidelines used by the MH [7-9]. The guidelines recommend the collection of key clinical data as determined by best practice evidence levels A or B. The guidelines were informed by Systematic reviews, National Institute for Health Care and Excellence (NICE) guidelines and relevant Australian guidelines, such as the National Health and Medical Research Council (NHMRC); Australasian Diabetes in Pregnancy Society (ADIPS); MH’s Antenatal Guidelines and New South Wales (NSW) Department of Health [9-14]. Recommendations were based on evidence about the accuracy of assessments in predicting complications in pregnancy and the effectiveness of interventions in reducing symptoms as described in Table 1.

**Table 1:** **Description of grades of recommendations from Clinical Practice Guidelines Antenatal Care-Module 1. (8)**

**Description**	**Grade**
Body of evidence can be trusted to guide practice	A
Body of evidence can be trusted to guide practice in most situations	B
Body of evidence provides some support for recommendation (s) but care should be taken in its application	C
Body of evidence is weak and recommendation must be applied with caution	D
Recommendation formulated in the absence of quality evidence (where a systematic review of the evidence was conducted as part of the search strategy)	CBR*
Area is beyond the scope of the systematic literature review and advice was developed by the EAC and/or the Working Group for Aboriginal and Torres Strait Islander Women’s Antenatal Care	PP**

*CBR-recommendation formulated in the absence of quality evidence (where a systematic review of the evidence was conducted as part of the search strategy.

**PP-Area is beyond the scope of the systematic literature review and advice was developed by the EAC and/or the Working Group for Aboriginal and Torres Strait Islander Women’s Antenatal Care.

In Phase 1 there were a total of 31 best practice variables identified as important from the guideline documents (see Table 2). Prior to Phase 2 data collection the Australian National Antenatal Guideline was updated and the evidence level of 2 variables changed. Iodine supplement advice and Vitamin D assessment were now categorised to the Level CBR (recommendation formulated in the absence of quality evidence (where a systematic review of the evidence was conducted as part of the search strategy)) and removed from the analysis. Additionally, ADIPS guidelines were revised and while in Phase 1 both GCT (glucose challenge test) and GTT (glucose tolerance test) were collected, in Phase 2 only GTT was required [13]. Expert consultation was sought to determine inclusion or exclusion where variables of importance were informed by relevant guidelines but did not have a specific evidence level attributed. These were morphology scanning, alcohol assessment, illicit drug use assessment and immunisation assessment of pertussis, hepatitis B and varicella. Consequently in Phase 2 there were a total of 28 variables identified as best practice. The final set of specific evidenced based best practice variables are shown in Table 2.

**Table 2:** **Specific best practice variables included in Phase 1 and Phase 2 from antenatal guidelines**

**Specific best practice variables**	**Evidence level (phase 1- draft guidelines)**	**Evidence level (phase 2-final guidelines)**
***Clinical measurements***		
BMI (body mass index)	*B*	*B*
Blood pressure	*B*	*B*
Proteinuria	*B*	*B*
***Screening***		
Blood group	*B*	*B*
Antibody status	*B*	*B*
Haemoglobin	*B*	*B*
Human immunodeficiency virus	*B*	*B*
Hepatitis B	*A*	*A*
Rubella	*B*	*B*
Syphilis	*B*	*B*
Urine culture (MSU)	*A*	*A*
GCT (glucose challenge test)	*ADIPS guidelines*	*Not included*
GTT (glucose tolerance test)	*ADIPS guidelines*	*ADIPS guidelines*
***Pregnancy assessments/advice***		
Dating scan	*B*	*B*
Nuchal scan	*B*	*B*
Morphology	*MH guidelines*	*MH guidelines*
Folic acid supplementation advice	*B*	*A*
Iron supplement advice	*B*	*B*
Use of vitamins in diet assessment	*B*	*B*
Iodine supplement advice	*NHMRC*	*CBR*
Vitamin D deficiency assessment	*B*	*CBR*
Oral health advice	*B*	*B*
Tobacco smoking	*B*	*A*
Alcohol assessment	*MH guidelines*	*MH guidelines*
Illicit drug use assessment	*MH guidelines*	*MH guidelines*
Domestic violence assessment	*B*	*B*
Mental health assessment (EDPS)	*NHMRC*	*B*
***Immunisation- pre-conception assessment - recorded***		
Pertussis	*NHMRC*	*NHMRC*
Hepatitis B	*NHMRC*	*NHMRC*
Varicella	*NHMRC*	*NHMRC*
***Immunisations required in pregnancy - recorded***	*NHMRC*	*A*
Fluvax		
	***n = 31***	***n = 28***

The primary outcome measure for the study was a composite score that consisted of all the best practice variables from the PHR and was measured out of 31 (in Phase 1) and in the EHR was out of 28 (in Phase 2). The secondary outcome measures were each of the best practice variables from the PHR (in Phase 1) and the EHR (in Phase 2).

### Procedure

The data collected for both the PHR and the EHR were predominantly collected at the first antenatal visit (exceptions are GCT and GTT). The completeness of data available in the PHR (in Phase 1) was assessed by auditing a sample of medical records (in which PHRs are filed) from pregnant women. One hundred charts were sampled from a possible 641 women who had participated in GP shared-care using a random number generating sequence in Excel. Data for Phase 2 of the study were extracted from the MH obstetric database selected from a sample of 732 potential women. Both samples were selected randomly using an excel spreadsheet function.

Data were recorded in an audit spreadsheet structured to capture the specific best practice variables, described in Table 3. Each variable was operationalised as ‘present’ or ‘not present’ and are shown in Additional file 2.

**Table 3:** **Description of best practice variables and timing of collection**

***Best practice variable***	***Description***	***Data collection time***
*Body mass index (BMI)*	Measure weight and height and calculate body mass index (BMI).	At first antenatal visit
*Blood pressure*	Measure blood pressure to identify existing high blood pressure.	At first antenatal visit
*Proteinuria*	Use an automated analyser if available, or urinary dipstick as less accurate method to detect true proteinuria.	At first antenatal visit or subsequent visits
*Blood Group*	Important to prevent haemolytic disease of the newborn	At first antenatal visit
*Antibody status*	As above	At first antenatal visit
*Haemoglobin*	To assess anaemia	At first antenatal visit
*Human immunodeficiency virus(HIV)*	Offer and recommend HIV testing	At first antenatal visit
*Hep B*	Offer and recommend hepatitis B virus testing.	At first antenatal visit
*Rubella*	Offer and recommend testing for rubella immunity	At first antenatal visit
*Syphilis*	Offer and recommend syphilis testing	At first antenatal visit
*Urine Culture (MSU)*	Use urine culture testing wherever possible as it is the most accurate means of detecting asymptomatic bacteriuria.	At first antenatal visit or subsequent visits
Glucose challenge test (GCT)	To screen for diabetes in pregnancy	Measured at 26–26 week visit
*Glucose tolerance test (GTT)*	To screen for diabetes in pregnancy	Measured at 26–26 week visit
*Dating scan*	Offer an ultrasound scan to determine gestational age, detect multiple pregnancies and accurately time fetal anomaly screening.	between 8 weeks 0 days and 13 weeks 6 days
*Nuchal translucency scan*	Offer nuchal translucency thickness ultrasound scan	Between 11 weeks 0 days and 13 weeks 6 days.
*Morphology*	To check for abnormalities in your baby.	Scan at 18–20 week gestation
*Folic acid supplementation advice*	Inform women of/determine if dietary supplementation with folic acid, from 12 weeks before conception and throughout the first 12 weeks of pregnancy occurred	At first antenatal visit
*Iron supplement advice*	Do not routinely offer iron supplementation to women during pregnancy.	At first antenatal visit
*Vitamin D deficiency*	Offer vitamin D screening to women with limited exposure to sunlight, have dark skin or a pre-pregnancy BMI of >30.	At first antenatal visit
*Oral health*	Advise/ask about oral health checks and treatment.	At first antenatal visit
*Tobacco smoking*	Assess the woman’s smoking status and exposure to passive smoking.	At first antenatal visit
*Alcohol*	Advise women who are pregnant or planning a pregnancy that not drinking is the safest option. Discuss alcohol consumed during pregnancy.	At first antenatal visit
*Drug Use- Illicit assessment*	Determine if ever used illicit drugs or requires assistance.	At first antenatal visit
*Domestic violence assessment*	Explain to all women that asking about domestic violence is a routine part of antenatal care.	At first antenatal visit

### Data analysis

The sample size was calculated based on the primary outcome of this research. The calculation was based on evidence found in the literature, reporting on completeness of health record data and in consultation with the MH statistician. Based on literature results of five non-maternity [15-19] and three maternity papers [20-22], it was assumed that 75% of records would be complete in Phase 1 and 90% of records would be complete in Phase 2. Considering a relative change of between records and using a 95% confidence interval, 97 records were needed in each phase of the study to detect a significant difference in the primary outcome. Data were analysed using SPSS for Windows (Version 21). Descriptive data analysis was undertaken using frequencies summarised using numbers and percentages. Pearson two-sided chi-squared analyses (or Fishers Exact tests for cell sizes less than 5) were planned to compare differences between the PHR and EHR frequencies. An alpha level of 0.05 was used to detect statistical significance.

### Ethical clearance

Low and negligible risk ethics clearance was granted from the Mater Health Services (LNR 1780QA). The Hospital's Privacy Office approved access to the hospital records following ethical clearance by the Mater Health Services' Human Research Ethics Committee.

## Results

Of the 100 medical charts audited in Phase 1 two charts were missing and four did not have a PHR filed within (PHRs are usually filed in the hospital chart at delivery) leaving a total of 94 charts available for audit. The number of missing PHRs were reported to the hospital’s data management team. In Phase 2 all records (100) were available from the obstetric database.

### Primary outcome

#### Completeness of data available from the PHR

From the expected total of 31 variables identified from the guidelines, 21 were recorded in designated fields in the PHR. Of the remaining ten variables, nine had results written in ‘free text areas’, or in areas with no prompt or question/answer space in the PHR, rather than in specific data fields (folic acid, iron supplement advice, vitamin supplement advice, vitamin D deficiency assessment, oral health advice, pre-conception evidence of pertussis, hepatitis B, varicella immunisations and ‘fluvax in pregnancy’ recommendation). There were no results for one variable (iodine intake advice) in either a designated field or in free text. Of the 31 specific best practice variables, none of the 94 women included in the chart audit had a complete dataset.

#### Completeness of data available from the EHR

In Phase 2 three best practice variables were not included in the composite score because they were no longer considered evidence level A or B in the National Clinical Practice Guidelines and changes were made to the ADIPS guidelines. These were GCT, iodine intake advice, vitamin D deficiency assessment). Of the 28 variables remaining relevant in Phase 2, there were 26 that had available fields present in the EHR. No EHR had a complete dataset.

In consultation with an MH statistician the chi-square analysis could not be performed, as neither Phase 1 or Phase 2 had a complete data set of best practice variables.

### Secondary outcomes

**Figure 1:**
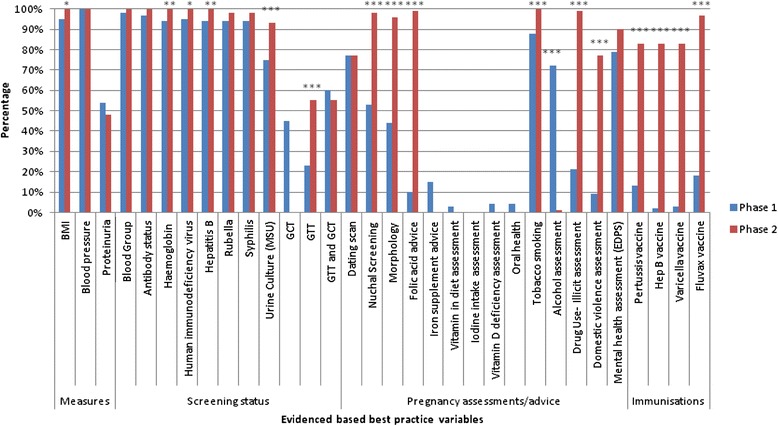
**Percentages of evidenced based best practice caribles between Phase 1 (PHR) and Phase 2 (EHR).** ***p ≤ 0.001, **p ≤ 0.01, *p ≤ 0.05 for comparisons between PHR and EHR MSU-Midstream urine, EPDS–Edinburgh postnatal depression scale, PHR-Paper hand-held record, EHR-Electronic health record.

Individual variables present in the PHR and EHR are shown in Figure 1, where differences in variable completeness are demonstrated between Phase 1 and Phase 2.

As shown in Figure 1, the only variable that did not have data recorded either in at specific data entry field or written in notes in the PHR was ‘iodine intake assessment’. Clinical measurements and screening results, excluding proteinuria, GCT and GTT were recorded within a range of 70-92%, as were dating scan, tobacco screening, alcohol assessment and mental health assessment (all >74%). The remaining variables of assessments/advice and immunisations were recorded poorly, with a variation between 3 and 51% (Figure 1). In Phase 2, EHR data were more complete. Clinical measurements and screening variables, excluding proteinuria, were recorded with between 93 and 100% completeness. Two variables from the total of 28 included in the analysis that did not have a data entry field in the EHR (iron supplementation advice and vitamin in diet assessment), consequently had no data recorded. Although there was a field for oral health, no data were available. During the introduction of the EHR, numerous changes were made to the alcohol assessment entry fields resulting in data not being recorded well. Recording of the assessments/pregnancy advice and immunisation variables (pertussis, hepatitis B, varicella, fluvax) were high with the EHR with a range of 77-100% completeness.

As demonstrated in Figure 1, the variables with improvement in completeness of documentation in the EHR, compared with the PHR were measures of urine culture and GTT (both *p = 0.001*). Similarly, recording of nuchal screening and morphology scans were significant (*p = 0.001*), as were folic acid advice, tobacco smoking, illicit drug assessment and domestic violence assessment *(p = 0.001).* The documentation of immunisations (pertussis, hepatitis B, varicella, fluvax) was markedly improved in the EHR (*p = 0.001*). The remaining variables were recorded as: BMI (*p = 0.02*), haemoglobin (*p = 0.01*), human immunodeficiency virus (*p = 0.02*) and hepatitis B status (*p = 0.01*).

The variables of GCT, iron supplementation, iodine intake assessment, and oral health were not compared for data completeness between the records. When GCT and GTT were combined to ascertain if variances existed due to the change in guidelines, no significant differences were found in data completeness between the PHR and EHR. Across both the PHR and EHR, there were no statistical differences between the clinical measurements of blood pressure, proteinuria, blood group, antibody status, rubella or syphilis. The assessment of mental health (using the Edinburgh depression scale) also demonstrated no differences in the recording between the records for the completeness of clinical measurements.

## Discussion

The data presented provide new insights into the availability of data recorded regarding documentation of care delivered to women in a GP shared-care maternity environment. This paper reports on the completeness of recorded specific best practice variables in a PHR, using counts and frequencies. Neither the PHR nor the EHR had a complete recording of these variables reflecting an apparent lack of adherence to best practice antenatal care guidelines. However, the comparison between the two record types did demonstrate significant improvements in completeness of data captured when using an EHR.

Both the PHR and EHR captured information usually collected at the first hospital antenatal visit to varying degrees of completeness. However, it was noted during the chart audit that the PHR variables were entered in an indiscriminate way resulting in troublesome extraction of information (in key data fields within the PHR as well as in free text areas). Previous work has recognised the PHR as a valuable tool to share data between the pregnant woman and her health professionals [2], but this current study demonstrates gaps in the quality of important antenatal information that the record is designed to capture. In practice when information is missing, care providers are required to search alternate databases or telephone for results and/or repeat requests for tests. This is an inefficient use of time and resources and introduces the possibility of clinical errors.

The EHR design provides an overall improvement in completeness of documented antenatal records at scheduled visit times, with significant improvements in important assessment (such as antenatal scans, GTT data) and immunisation recorded. Overall, the EHR provides an avenue for all clinicians to access a more complete antenatal dataset, although the reliance on presence or availability of “data fields” to capture all best practice variables may be a short-coming if they are not programmed in to the system, as was evident in our study. However, the pattern of available data between the two records does suggest the mode of record keeping does influence the completeness of data captured.

In practice the EHR permits continuity of access to information between hospital and community providers. The EHR has the capacity for information to be available in real-time, to multiple users who can simultaneously view and enter data. This is particularly relevant in the shared-care setting, where the community GP provides care to woman at potentially ten antenatal visits, but may also be useful when allied health, midwifery and medical staff are all providing care for a woman during a single clinic visit. The introduction of the EHR in a GP shared-care maternity setting is integral to the roll-out of a National EHR in Australia [4]. The MH EHR has demonstrated an improvement in up to date, more complete, readily available and accessible information for hospital and community clinicians and the pregnant woman. This initiative is an important step in increasing access to high quality clinical information and integrating care between maternity care providers and women.

A strength of this study was the utilisation of relevant practice guidelines on which to examine the quality of the PHR and EHR. The guidelines referred to in this study are used to assist practitioners to make appropriate health care decisions in different circumstances in a GP setting, but also to ensure uniformity and reliability of clinical data [23]. Some limitations were evident; despite undertaking power calculations to ensure we reviewed sufficient records, few studies existed from which to draw these informative statistics. The introduction of the EHR did bring about discrepancies in data entered due to changes in data entry labels as seen in the alcohol assessment field. This was recognised and accounted for, although analysis of the recording of this variable could not be considered an accurate assessment of alcohol consumed. Also, the update of national guidelines did result in adjustments of variables included and subsequent adjustments were made to the denominators in the analysis. An additional aspect of the study demonstrated that while the data were extracted based on relevance according to guidelines, there was no consultation with women to gain a perspective on the requirements they would like to see in a maternity EHR. Further research into preferred personal access by pregnant women would give more insight into completing the picture of information important in a GP shared-care setting [2]. Additionally while data were drawn from specific fields in the EHR, this prevented access to the ‘free text’ sections that were added to some of the variable data entry fields, whereas it was possible to find some of the best practice variables written in freehand locations, as noted in the PHR audit. A further limitation to introducing and optimising usage of an EHR is facilitating and enabling clinicians to have access to e-health technology. Additionally, an EHR will only be useful and have maximum potential if hospital clinicians, GPs and pregnant women decide to adopt it.

## Conclusions

The PHR is a popular record keeping tool that is widely accepted by women and health care providers to document antenatal information across women, hospital and community. In alignment with the introduction of the National EHR, a South East Queensland (Australia) MH has implemented an EHR to share data between its clinicians and GPs involved in maternity shared-care. While neither record resulted in complete capture of all required best practice variables, use of an EHR demonstrated improved access to antenatal clinical information and greater adherence to the collection of these variables. While the PHR does record best practice variables, many of these are difficult to locate in a free text form and only retrospectively found by an audit process. The EHR has the capacity to further improve data capture by ensuring there are specific fields in which to enter an increased number of best practice variables. This study adds to an under-researched, but important area of clinical data quality and is the first step in determining how to improve recording of complete, relevant and up to date antenatal information that can be shared between maternity health care providers and women. The experiences of health care providers and of women using the maternity records is also of interest in this program of research and will be addressed in future papers.

## Additional files

Additional file 1:
**Comparison of features of PHR and EHR data systems.**
Additional file 2:
**Operationalisation of variables.**

## Abbreviations

PHR: Paper hand-held record
EHR: Electronic health record
GP: General practitioner
MH: Maternity Health
