# Supplementary material for: Sharing of clinical data in a maternity setting: How do paper hand-held records and electronic health records compare for completeness?
Source: BMC Health Serv Res. 2014 Dec 21;14:650. doi: 10.1186/s12913-014-0650-x (PMC4302146; doi:10.1186/s12913-014-0650-x)
Supplement: Additional file 1: — Comparison of features of PHR and EHR data systems. [file 12913_2014_650_MOESM1_ESM.docx]

**Additional File 1.** Features of the PHR and EHR

| **PHR – Paper hand-held record** | | **EHR – Electronic health record** | | |
| --- | --- | --- | --- | --- |
| ***Pages are divided into sections of:*** | | ***Icons viewed through patient portal:*** | | |
|  |  | Information entered into EHR by health care providers | | |
| **Pages 1 to 3** | *Mother and general practitioner details* | **Antenatal history** | *History recorded early in pregnancy* | |
| **Pages 4 to 5** | *Important antenatal signs and symptoms of concern*  *Birth preferences* | **Issues and plans** | *Identified medical and obstetric issues and management plans* | |
| **Pages 6 to 7** | *Baby feeding intentions, glossary and what to bring to hospital, additional notes section* | **Healthcare providers** | *Details about the providers of maternal care* | |
| **Pages 8 to 9** | *Antenatal visit schedule and care checklist* | **Antenatal visits** | *Summaries of visits to clinicians for antenatal care (these fields do not allow you to progress without a response and include: allergies, smoking/alcohol and drugs, domestic violence and mental health assessment)* | |
| **Pages 10 to 11** | *Father and mother health history*  *Previous pregnancy information* | **Test results** | *Results of laboratory and ultrasound tests (these fields do not allow you to progress without a response and include screening of: blood group, rhesus factor, antibodies, haemoglobin, HIV, Rubella, Syphilis, Hepatitis B, Urine culture, Glucose tolerance test, Nuchal and morphology scanning).* | |
| **Pages 12 to 13** | *Laboratory and ultrasound results*  *Medical and obstetric issues and management plans* | **Reports** | *Pregnancy reports to view and print* | |
| **Pages 14 to 17** | *Fundal height chart*  *Visit notes* | Details recorded by women | | |
| **Pages 18 to 20** | *Tobacco and alcohol screening*  *Additional scheduling section* | **Notes/questions** | | *To record my notes and questions for providers* |
|  |  | **Birth preferences** | | *Preferences for birth and postnatal care* |
